# *Haematococcus pluvialis* bionanoparticles boost maize seedling health, serving as a sustainable seed priming agent and biostimulant for agriculture

**DOI:** 10.1111/ppl.70245

**Published:** 2025-05-01

**Authors:** Nahid Rafiei, Hossein Alishah Aratboni, Leandro Luis Lavandosque, Clíssia Barboza Mastrangelo, Welinton Yoshio Hirai, Lucianne Ferreira Paes de Oliveira, Gabriel Luiz Padoan Gonçalves, José Lavres, Mônica Lanzoni Rossi, Adriana Pinheiro Martinelli, Simone Possedente de Lira, Seyed Abdolreza Kazemeini, Flavia Vischi Winck

**Affiliations:** ^1^ Laboratory of Regulatory Systems Biology, Center for Nuclear Energy in Agriculture University of São Paulo São Paulo Brazil; ^2^ Laboratory of Radiobiology and Environment, Center for Nuclear Energy in Agriculture (CENA) University of São Paulo (USP) São Paulo Brazil; ^3^ Department of Exact Sciences University of São Paulo, Luiz de Queiroz College of Agriculture (USP/ESALQ) São Paulo Brazil; ^4^ Laboratory of Stable Isotopes, Center for Nuclear Energy in Agriculture University of São Paulo São Paulo Brazil; ^5^ Laboratory of Plant Biotechnology, Center for Nuclear Energy in Agriculture University of São Paulo São Paulo Brazil; ^6^ Department of Plant Production and Genetics, School of Agriculture Shiraz University Shiraz Iran

## Abstract

The rising frequency of extreme climate events requires sustainable strategies to secure food production. Environmental stress impacts seed germination and seedling development, posing a significant agricultural challenge. To address this, we developed and applied iron‐based nanoparticles (Bio‐NPs) synthesized through green biosynthesis from *Haematococcus pluvialis*, a microalga rich in antioxidants like astaxanthin. These Bio‐NPs, approximately 21 nm in diameter and characterized by a negative surface charge, were used as priming agents for maize seeds. Their effects on physiological traits were analyzed with multispectral imaging, showing enhanced normalized difference vegetation index (NDVI) and chlorophyll levels in maize seedlings, highlighting Bio‐NPs as effective biostimulants.

Among the tested concentrations, 6 mM Bio‐NPs yielded the most substantial improvements in seedling health compared to unprimed and hydro‐primed groups. Importantly, in vitro studies confirmed that Bio‐NPs had no harmful effects on beneficial bacteria and fungi of agronomic importance, underscoring their safety. Although the exact biological pathways responsible for these enhancements are yet to be fully understood, further research into plant responses to Bio‐NPs could yield new insights into plant biostimulation.

Bio‐NPs thus hold promises for strengthening seedling resilience under extreme environmental scenarios, currently observed due to global climate change, offering a safe, sustainable approach to agricultural enhancement. By leveraging microalgae‐based biostimulants, this work advances seed priming technology, fostering crop resilience and supporting environmentally friendly agricultural practices.

## 1 INTRODUCTION

Environmental stresses, particularly drought, salinity, and extreme temperatures, are the most significant factors restricting seed germination and seedling development (Walck et al., 2011; Reed et al., 2022). To mitigate the effects of biotic or abiotic stresses on initial plant growth, seed metabolism can be modulated by priming techniques (do Espirito Santo Pereira et al., 2021; Hussain et al., 2019). Seed germination is a critical biological process that strongly influences the plant's entire growth cycle. Successful germination positively affects plant establishment, survival, and yield, especially under less favorable conditions (Makhaye et al., 2021). Seed germination is typically divided into three phases: seed imbibition (water uptake), reactivation of metabolic pathways, and the emergence of the embryonic axis, usually the radicle (Makhaye et al., 2021).

Seed priming, a pre‐sowing seed treatment, activates pre‐germinative metabolism while preventing radical protrusion through the seed coat (Tekrony, 2001). It has been shown to accelerate the germination process and improve uniformity, addressing aspects of low seed vigor and poor product quality (Chatterjee et al., 2018; Devika et al., 2021; Zulfiqar, 2021). Therefore, in contrast to unprimed seeds, primed seeds usually show higher germination rates and more uniform emergence, with potential improvements in plant memory toward higher environmental resilience (Chen and Arora, 2013; Abid et al., 2018).

Several possible seed priming agents have been applied in agricultural practices, looking to improve plant growth, development and yield by enhancing plant's resilience during germination, the most vulnerable stage of its development. Seed treatments can be performed by physical, chemical or biological means with the aim to provide increased plant growth and accelerated field emergence but also to improve plant resistance to pests. Chemical compounds that function in the control of diseases (e.g. Carboxin, Captan, Maneb) and osmopriming agents (e.g., calcium chloride (CaCl_2_), heat treatment (water heat, aerated heat or dry heat), and biological compounds such as metabolites including hormones [e.g., melatonin, abscisic acid (ABA) and brassinosteroid (BR)], and inoculation with bacterial cultures (e.g., Trichoderma spp., Azospirillum, and Pseudomonas fluorescens) have previously been applied in seed treatments, improving plant emergence and production (Sharma et al., 2015).

However, other recent applications of seed priming include the application of nanomaterials, showing improvements in seed germination and seedling development, improving also plant performance under abiotic and biotic stresses. Usually, seed priming with nanoparticles (nano‐priming) is performed with minerals (Co, Zn, Fe) and metal‐based (Co, Mn, Cu, Fe, Zn, Mo and Se) materials, which have demonstrated to improve seed germination (Fu et al., 2024).

Metallic nanoparticles (NPs), such as zinc oxide, iron oxide, and gold‐based NPs, have been described as effective seed‐priming agents, promoting plant growth and reducing oxidative stress (Rizwan et al., 2019). Furthermore, previous studies revealed that NPs synthesis using chemical‐based methods, which applies a strong base (e.g. NaOH, ammonium hydroxide, and CH3NH2) as a reducing agent (Ali et al., 2016), could be substituted by methods using biological compounds (e.g. plant and microalgae extracts) in the synthesis reactions (Arteaga‐Castrejon et al., 2024). Therefore, the green synthesis of NPs has become a non‐toxic and environment‐friendly alternative method (Nitnavare et al., 2022b). In addition, the application of NPs as plant biostimulants has demonstrated their potential to stimulate plant growth, and their physicochemical properties, such as energy and surface charges, seem to be relevant in their effects on the treated seeds and plants (Juarez‐Maldonado et al., 2019).

The chemical composition of NPs, along with the combination of their inner and outer compounds, determines their biostimulant capacities and effects on plants, modulating the cell wall and membrane, eliciting various plant responses (Juarez‐Maldonado et al., 2019). The contact or uptake of metal nanoparticles may elicit oxidative stress intracellularly in treated plants since their chemical oxidative status may change once under physiological conditions. Therefore, the physiological responses observed in plants may be associated with the presence of NPs itself over the seed's surface or as the result of the activation of a plant cellular mechanism eliciting a stress response in a non‐destructive way, such as hormesis (Godínez‐Mendoza et al., 2023).

The presence of transition metals, including iron (Fe), copper (Cu), chromium (Cr), to mention a few, are involved in the generation of Reactive Oxygen Species (ROS) in reaction mechanisms of Haber‐Weiss and Fenton‐type reactions, yielding several molecules, including OH˙, O_2_˙^−^, OH˙, activating a prooxidant effect in cells (Manke et al., 2013). In plants, non‐toxic stimulation of ROS may stimulate a low level of stress response, which is able to boost plant defenses to abiotic and biotic stressors without compromising plant growth (Godínez‐Mendoza et al., 2023).

However, the use of nanoparticles alone may bring some environmental risks associated with the associated pollution from their chemical synthesis processes and their possible environmental toxicity (Fu et al., 2024).

Therefore, the development of green synthesis of bionanoparticles using organic molecules from plant or microalgae extracts as reducing agents in nanoparticle's synthesis renders a more environmentally friendly application combining the benefits of nano‐priming with the delivery of beneficial organic molecules to the seeds.

The application of microorganisms as seed‐priming compounds enhances plant growth, improves nutrient uptake, and increases plant resilience to abiotic stress (Cardarelli et al., 2022). It is expected that each novel combination of NPs and surface compounds may elicit different molecular responses in plants, and further studies are required to investigate their effects on various plant species, especially under varying environmental conditions.

Thus, microalgae biomass rich in bioactive molecules, such as specialized metabolites, proteins, carbohydrates, and pigments, are potential sources to produce NPs (Nitnavare et al., 2022a). Their ability to grow in wastewater (Nitnavare et al., 2022a; Alishah Aratboni et al., 2019; Naseema Rasheed et al., 2023; Chaudhary et al., 2020) and a great potential for accumulating astaxanthin, a keto‐carotenoid pigment with antioxidant properties, makes species like *Haematococcus pluvialis*, a unicellular microalga (Oslan et al., 2021; Shah et al., 2016), a rich source of biomolecules to act as complex reagents for the biosynthesis of metallic NPs, contributing to achieving circular and green economy goals.

The combination of microalgae extract into nanoparticles synthesis could bring a novel, more stable and safer use of macromolecules from microalgae extract by not delivering intact cells to the field, leading to lower environmental impact compared to traditional agrochemicals and synthetic products. The application of microalgae biomass cultivated under controlled and industrial conditions should have a higher biomass compositional reproducibility and safety than the application of macroalgae extracts and biomass harvested from the oceans into agricultural practices.

Several beneficial effects of microalgae fresh biomass or extracts as biostimulants have already been described for several plants, including Lettuce, Broccoli, Tomato, Sugar Beet, and results are variable depending on application method and plant variety (Chabili et al., 2024).

In contrast to the application of microalgae biomass produced under controlled conditions, it has been previously observed that environmental brown algae have an interspecific and temporal variation in the accumulation of phenolic compounds in its natural environment, which may change from annual seasons and even with the depth of the water column, affecting their biomass composition (Connan et al., 2004).

In other macroalgae species already applied in agronomic products, including *Ascophyllum nodosum*, the phenolic pools and capacity to accumulate metals such as zinc, cadmium and copper, were modified by the environment salinity, which could change the amount of total content of accumulated metals in the macroalgae biomass (Connan et al., 2004).

In fact, algae are the main class of organisms that excel in terms of heavy metals uptake and store, which may contribute to their uptake of a large proportion of heavy metals present in lakes and ocean. New regulations towards the registration of new biofertilizers and biostimulants have been imposed in some countries towards collecting evidence of their chemical safety, especially related to heavy metals, before any agronomic application of algae biomass (Souza et al., 2012, Regulation (EU) 2019/1009).

In this context, our study aimed to develop a fast, feasible, and eco‐friendly method for the biosynthesis of NPs based on *H. pluvialis* extract. This microalgae species has already been described as an industrial strain with several potential applications in the food and pharmaceutical industry due to its biochemical characteristic of astaxanthin accumulation. This microalga can be cultivated under controlled, contained and large‐scale operations, providing an important source of bioproducts (Shah et al., 2016).

Therefore, it is desirable to develop sustainable and controlled applications for microalgae biomass, combining the known benefits of the microalgae biomass and the important characteristics of nanoparticles to plant nutrition, adding value to novel biostimulants.

We investigated their biostimulant effect in maize (*Zea mays* L.) through seed priming and evaluated the physiological responses of seedlings and potential impacts on agriculturally important microorganisms. Maize seed priming has already been addressed as a way to improve productivity using zinc and cow urine (Dawadi et al., 2023). However, to our knowledge, no application has been performed using nanoparticles developed from the microalgae *H. pluvialis* towards seed priming in maize plants. The use of multispectral imaging (Bianchini et al., 2021; Barboza da Silva et al., 2021) allowed for detailed analysis of plant health and chlorophyll content. Further analysis of Bio‐NPs on the growth of selected microorganisms and as a biostimulant in maize confirmed the potential environmental compatibility of these Bio‐NPs in modern agricultural applications.

With this approach, we investigated the feasibility of synthesizing and applying microalgae‐based nanoparticles as an environmentally friendly plant biostimulant and seed priming agent. The investigation of the effects of these Bio‐NPs on maize seedlings improved our understanding of the possible agronomic benefits this approach could bring to modern agriculture, especially related to the enhancement of plant resilience in a climate change scenario.

## 2 MATERIALS AND METHODS

### 2.1 Preparation of *Haematococcus pluvialis* extract

*H. pluvialis* extract was prepared from 1600 mL cell culture with the primary concentration of 1x 10^5^ cells mL^−1^ cultivated in Tris‐Acetate‐Phosphate (TAP) medium for seven days at 25°C under continuous light (~100 μmol m^−2^ s^−1^) and constant orbital agitation of 100 rpm (Infors HT Multitron Pro Incubator Shaker). Afterward, under aseptic conditions, the culture was transferred to suitable sterile centrifuge tubes and were centrifuged for 10 min at 2500 x *g* (Eppendorf Centrifuge 5810R). The cell pellets were suspended in the same volume of TAP medium without the nitrogen source (NH_4_Cl). The applied nitrogen starvation is known to promote the accumulation of astaxanthin in the cells, and subsequently, the color of the cell culture changed from green to red‐brownish. After two weeks, the culture was centrifuged as described above, and the cell pellets were rinsed twice with deionized water. The cell pellet was dissolved in 130 mL deionized water, heated at 60°C for 15 min., and filtered using qualitative filter paper (Whatman filter paper 10 mm) to obtain the final microalgal extract.

### 2.2 Synthesis of Bio‐nanoparticles

The synthesis of the Bio‐NPs was performed using 4 g of iron chloride (III) (FeCl_3_) and 2 g of iron chloride (II) (FeCl_2_) dissolved in 400 mL of deionized water in a sterile Erlenmeyer flask with a closed lid, and under continuous stirring on a heating plate, until the reaction temperature reached 80°C, which was maintained for 3 h. Microalgae extract (10 mL) was added to the prepared iron solution under continuous stirring. After 13 min, the dispersion pH was adjusted to 12 with a 5 mol L^−1^ sodium hydroxide (NaOH) solution. During the pH adjustment, the color of the iron dispersion with microalgae extracts gradually changed from dark yellow to black, which is the first confirmation of the Bio‐NPs synthesis. Then, the Bio‐NPs were collected by centrifugation at 12.000 x *g* for 30 min and rinsed twice with deionized water. The synthesized Bio‐NPs were then dried at 80°C for 24 h until further use.

### 2.3 Spectroscopic and microscopic characterization of size distribution, surface charge, crystalline structure, and morphology of the synthesized Bio‐NPs

The UV–Vis absorbance spectrum of the Bio‐NPs was obtained in water using a UV–Vis spectrophotometer (TECAN Infinite 200 Pro) and processed with compatible software (Tecan i‐control, 2.0.10.0) to determine their absorption peak between 295–301 nm, a characteristic evidence of the iron nanoparticle synthesis. Furthermore, the average size of Bio‐NPs, particle size distribution, and morphological characteristics were analyzed by spotting a drop of Bio‐NPs suspension over a carbon grid, which was dried at room temperature, and the structures were examined, identified and confirmed by transmission electron microscopy (TEM) observations. Bio‐NPs solution was centrifuged at 12,000 x *g* for 30 min., rinsed twice with deionized water, and a small droplet of the solution was deposited on formvar/carbon supported copper grids (200 mesh), dried at room temperature for 24 h, and observed under a TEM at 120 kV (JEM1400 JEOL). These analyses were performed to confirm the nanometric structures (Ali et al., 2016) with the expected spherical shape and negative charge usually observed in iron nanoparticles (Yousif et al., 2023).

The X‐ray Diffraction (XRD) analysis (Rigaku, Japan) was performed to characterize the crystalline structure of the Bio‐NPs. The Bio‐NPs suspension was analyzed using background subtraction with Sonnevelt‐Visser's method, with maximum width of peak of 1.500 degrees, minimum height of peak of 0.5 cps, and peak search using 2nd differential method, typical width of 0.5 degrees and minimum height of 50.00 cps. Zeta potential measurements were carried out using Zetasizer Nano instrument with software version 8.02 (Malvern) analyzing the surface charge and stability of the synthesized colloidal Fe_3_O_4_NPs and Bio‐NPs. The apparent Zeta potential was measured with the system at 25°C, 135.8 kcps, 12 Zeta runs, in water as dispersant.

In addition, the nanoparticle's efficiency and iron oxide incorporation into the nanostructures were evaluated by comparing the iron content in the original iron solution and the remaining iron content in the supernatant of nanoparticles and Bio‐NPs suspension. The analysis was performed using Flame Atomic Absorption Spectroscopy (FAAS) with a AA240FS fast sequential atomic absorption spectrometer (Varian), and the data analyzed using the Spectra Worksheet Oriented Software, 5.1 PRO Version, following the manufacturer's instructions for iron analysis.

### 2.4 Evaluation of Bio‐NPs effects on maize seed germination and seedling physiological parameters using multispectral imaging analysis

Maize seeds were primed by soaking in several concentrations of Bio‐NPs dispersion (2 mM, 4 mM, 6 mM, 8 mM, and 16 mM) for 20 h and stirring at 150 rpm. The soaking duration was defined as the period until no further water uptake was detected by weight measurements. The seeds under hydro‐primed (HP) condition were disinfected using 0.1% sodium hypochlorite, rinsed, and primed with distilled water in the same manner. The control consisted of unprimed (UP) seeds that did not receive any priming treatment. The concentrations applied in our study were chosen based on previous studies of the effects of iron nanoparticles application in *Eruca sativa*, performed in similar range of iron concentrations (Plaksenkova et al., 2019).

Primed seeds were air‐dried for 48 h and then analyzed by multispectral and fluorescence‐based techniques using VideometerLab4™ (Videometer A/S) and SeedReporter™ (PhenoVation B.V.) instruments to investigate their reflectance and pigments as previously described (Fonseca de Oliveira et al., 2022).

Twenty‐five seeds per treatment condition and control HP and UP seeds were sown on Germitest paper moistened with distilled water [1:2.5 (w/v)] and carefully rolled. Plants from UP, HP and 4 mM treatment were also germinated on sterilized sand and observed during germination and emergence from soil. Four experimental replicates of each treatment were performed and kept at 25°C with an 8 h photoperiod. On the 7th day after sowing, the seedlings were evaluated for several physiological parameters and reflectance in several spectral wavelengths using the SeedReporter™ instrument. Images of the plants cultivated in sand were taken 7 days after sowing.

### 2.5 Multispectral image analysis of spectral parameters of seeds and seedlings

The VideometerLab4™ (Videometer A/S) and SeedReporter™ (PhenoVation B.V.) devices registered the reflectance data from the seeds. The reflectance images from maize seeds were captured at 19 different wavelengths (365, 405, 430, 450, 470, 490, 515, 540, 570, 590, 630, 645, 660, 690, 780, 850, 880, 940, and 970 nm). Multispectral images were captured for all treatments and replicates. The results were extracted using VideometerLab software version 3.14.9.

SeedReporter™ equipment, which offers a LED‐based lighting system, is a rapid, non‐destructive, and accurate technology for measuring chlorophyll *a* (Chl/a) fluorescence, anthocyanin Index (AriIdx), and chlorophyll *a* Index (ChlIdx), based on fluorescence and spectral reflectance images. Light intensity was adjusted before image capturing, and in a few seconds, the fluorescence and reflectance images were captured, which resulted in multispectral images with a spatial resolution of 2448×2448 pixels (3.69 m/pixel). Plants were exposed to white light (3000 k) in the wavelenght from 450 to 780 nm and reflectance data were collected using four optical wavelength filters (540, 640, 710, and 770 nm). The chlorophyll *a* index was estimated using data reflectance obtained at 710 and 770 nm (Gitelson et al., 2003) and the AriIdx calculated using reflectance signal obtained at 540, 710, and 770 nm (Gitelson et al., 2001). NDVI was calculated based on the mathematical model proposed by Rouse and Haas (1973) using spectral reflectance patterns of leaves at 640 nm (red) and 770 nm (near‐infrared) wavelengths (Rouse et al., 1973). This ratio represents the relative amount of light reflecting from leaves at both wavelengths.

The following formulas were used by the SeedReporter™ software to calculate the ChlIdx (Gitelson et al., 2003), AriIdx (Gitelson et al., 2001), NDVI (Rouse et al., 1973) parameters, where spectral reflectance for each respective wavelength is represented by ρ (Equations (1), (2), (3)):

(1) $$\text{Chlorophyll}\;a\;\text{Index}=\left(\frac{\rho_{770}}{\rho_{710}}\right)-1$$

(2) $$\text{Anthocyanin Index}=\rho_{770}\left(\frac{1}{\rho_{540}}\right)-\left(\frac{1}{\rho_{710}}\right)$$

(3) $$\text{NDVI}=\left(\frac{\rho_{770-}\rho_{640}}{\rho_{770+}\rho_{640}}\right)$$

### 2.6 *In‐vitro* assay of bacterial growth response exposed to the Bio‐NPs

We performed a microbiological investigation of the effects of Bio‐NPs treatment on the growth of *Bacillus thuringiensis RZ2MS9* (Andrade et al., 2020), *Azospirillum brasiliense Ab‐V5* (de Almeida et al., 2021), and *Pantoea agglomerans 33.1* (Ferreira et al., 2008), which are microorganisms of known beneficial interactions in the agricultural systems. These bacterial species were cultured on Tryptic Soy Agar medium (TSB) and stock cultures were maintained on agar Luria‐Bertani (LB) medium. Fresh solid cultures were incubated at 37°C for 24 h and maintained in the same conditions to avoid media exchange. After 24 h, some bacterial colonies from axenic cultures were cultivated in 2 mL of Mueller‐Hinton Broth (MHB).

Preparation of the materials and antimicrobial susceptibility testing were done according to the Clinical and Laboratory Standards Institute (CLSI) proceedings (Wikler et al., 2018). Bacterial suspensions were adjusted to 0.5 McFarland turbidity standards through optical density measurement in 540 nm by UV–Vis spectrometer (FEMTO). Then, the antimicrobial activity of different concentrations of Bio‐NPs dispersion (4, 8, 16, 32, 128, and 256 μg mL^−1^) was evaluated using the microdilution method in a 96‐well culture plate (Teh et al., 2017), with these concentrations defined according to CLSI recommendations in the context of microorganism susceptibility (Humphries et al., 2021).

As a positive control, tetracycline was used as a broad‐spectrum antibiotic with the same concentrations of NPs. As a negative control, the different concentrations of the bacterial suspensions were adjusted to 0.5 McFarland in the absence of NPs or antibiotics. The cell suspensions were incubated for 24 h at 37°C, followed by measurements of the absorbance at 540 nm, using a plate‐reader spectrophotometer (TECAN). Afterward, resazurin solution (0.01%) was added to each well and samples were incubated for 2 h to evaluate the cell viability.

### 2.7 *In‐vitro* assay of fungal growth exposed to the Bio‐NPs

In this study, *in‐vitro* fungal response to Bio‐NPs presence was evaluated by the agar incorporation test. For this purpose, commercial *Trichoderma harzianum* (Commercial strain) served as a target species. A stock dispersion of 15300 μg mL^−1^ Bio‐NPs was prepared and sonicated. Potato dextrose agar (PDA) medium (Merck) was prepared (40 g L^−1^) and autoclaved. At about 50°C, five different concentrations of Bio‐NPs dispersion (125, 250, 500, 1000, and 2000 μg mL^−1^) were added to prepare different media concentrations from the nanoparticles stock, and PDA was immediately poured on culture plates. As a positive control, the iron salt solution used to prepare the Bio‐NPs solution was mixed in the PDA medium with a final concentration of 2000 μg mL^−1^. The negative control was a PDA medium without NPs or iron solution. The autoclaved medium was poured into 90x15 mm polystyrene disposable Petri dishes and incubated at 35°C for 24 h in the dark. Then, as previously described, a 5x5mm^2^ square of an active colony of *T. harzianum* was transferred to the center of the mentioned plates (Adhikari et al., 2018). They were incubated at 28°C in the dark until the fungi in the control plate grew their mycelium to cover all plate surfaces. After six days, the diameter of the colonies was measured using the ImageJ software (National Institutes of Health, USA). Plate imaging started on the third day of the experiment (3rd day of culture (DC)). The experiments were done with four biological replicates.

### 2.8 Statistical analysis and classification of multispectral data

Principal Component Analysis (PCA) method allows the interpretation of the variables through a two‐dimensional plot (biplot ‐ PC1 x PC2). It was applied to the multispectral image data of primed seeds with different concentrations of Bio‐NPs dispersion and their generated seedlings, separately. For this purpose, new variables were generated employing uncorrelated linear combinations (eigenvectors) of the original variables. For a better interpretation of the original variables, they were standardized, where each observation was weighted by subtracting the mean value and dividing it by the standard deviation of the respective variable (Shlens, 2014).

A Random Forest (RF) classifier based on the Gini index was applied to multispectral data to classify the data into clusters of most relevant characteristics. The RF method is a machine learning technique that enhances classification and regression tree models. In this case, multiple partitions of the data set were randomly generated computationally, and a classification model was adjusted for each partition (Ruppert, 2004). Each variable was removed from the simulations, the tree accuracy was calculated, and this process was randomly repeated several times. Data classification analysis using Random Forest applied 250 trees (ntree = 250) and cross‐validation was performed with the calculation of Accuracy and Kappa values using caret R package with 5‐fold cross‐validation, selecting the optimal model based on Accuracy values. Ultimately, a plot was generated to evaluate the important variables in the RF.

Pearson's correlation coefficient was calculated to evaluate the relationship between different reflectance data, which refer to the plant physiological parameters. Differences in parameters measured between treatments with Bio‐NPs were tested using Fisher's least significant difference (LSD) tests. PCA, RF, and Pearson's correlation analyses were performed using R version 4.2.2 (Team, 2014), and RStudio (RStudioTeam, 2020). The factoextra:: (Kassambara and Mundt, 2020), randomForest:: (Liaw and Wiener, 2002), and corrplot:: (Friendly, 2002) libraries were used for constructing PCA biplot, RF modeling, and correlation graphs, respectively. The LSD test was performed by SAS (version 9.1.3) statistical software.

## 3 RESULTS

The synthesis of iron oxide nanoparticles (Bio‐NPs) was successfully mediated by microalgae cell extract. The Bio‐NPs obtained were characterized based on their physico‐chemical and spectroscopic properties, and their effects on maize seeds and seedlings, resulting from seed priming treatments, were evaluated using multispectral imaging. Additionally, the potential toxic effects of the Bio‐NPs on various microorganisms were assessed.

### 3.1 Characterization of structure, charge, and size of synthesized Bio‐NPs

**FIGURE 1:**
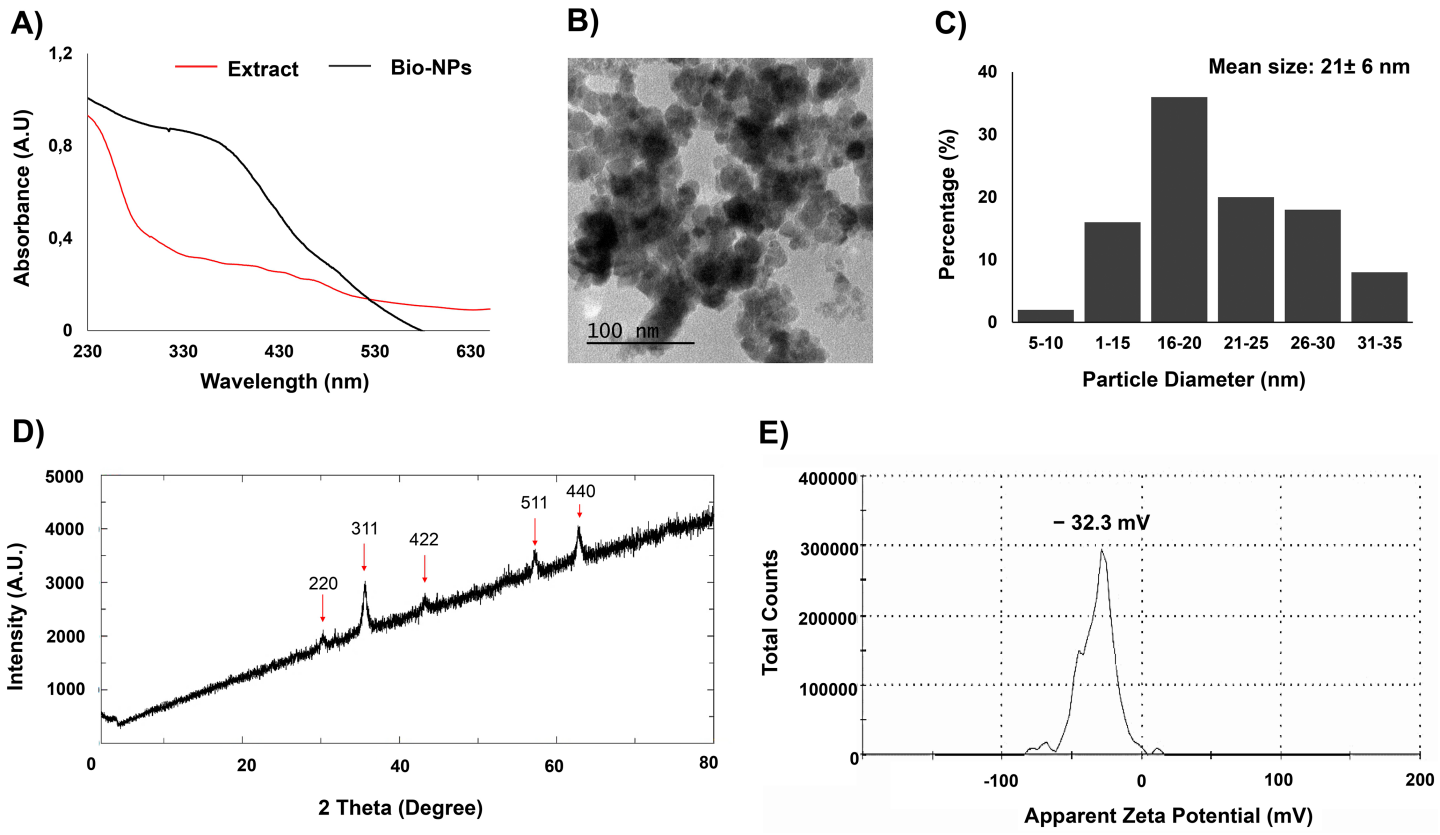
Spectroscopic and microscopic characterization of synthesized Bio‐NPs. (A) Surface plasmon light absorption of Bio‐NPs (black curve) with a maximum absorbance at 316 nm, compared to the microalgae extract (red curve); (B) transmission electron microscopy (TEM) micrograph of Bio‐NPs, showing their spherical morphology; (C) Histogram showing the particle size distribution of Bio‐NPs based on TEM measurements; (D) X‐ray diffraction pattern with diffraction peaks at 2θ values of 30.26, 35.60, 43.19, 57.26, and 62.77, corresponding to the crystal structure of Bio‐NPs; (E) The Zeta potential measurement of synthesized Bio‐NPs indicating a negative charge of −32.3 mV.

Initially, the Bio‐NPs synthesis was monitored using UV–Vis light absorption. While the microalgae extract alone showed no significant absorption peak, the Bio‐NPs exhibited their maximum absorption peak at 316 nm, indicating successful nanoparticle formation (Figure 1A).

Transmission electron microscopy (TEM) analysis revealed the predominant spherical morphology and indicated the presence of a diffuse, possibly organic outer thin layer, likely derived from the *H. pluvialis* extract surrounding the Bio‐NPs (Figure 1B). According to the particle size distribution obtained from TEM analysis, the average size of the Bio‐NPs was 21.14 nm (Figure 1C).

The crystalline structure of Bio‐NPs was characterized through X‐ray diffraction (XRD) (Figure 1D). The XRD spectrum showed diffraction peaks at 2θ of 30.26, 35.60, 43.19, 57.26, and 62.77, corresponding to (220), (311), (422), (511), and (440) crystal planes of a pure Fe_3_O_4_ (Yusmaniar et al., 2017) with the inverse cubic spinel phase, confirming the successful biosynthesis of Bio‐NPs (Zhang et al., 2013). The measured zeta potential was found to be −32.3 mV, indicating that the synthesized Bio‐NPs are negatively charged (Figure 1E).

Flame Atomic Absorption Spectrometry (FAAS) analysis revealed that 99.998% of the initial amount of iron used in the original salt solution were converted into Bio‐NPs during the synthesis process, using the microalgae extract as a reducing agent. This high conversion rate demonstrates the efficiency of the biosynthesis method and the high potential of the *H. pluvialis* extract for producing bionanoparticles.

### 3.2 Multispectral imaging analysis: effects of the Bio‐NPs on spectral parameters of seeds and seedlings

**FIGURE 2:**
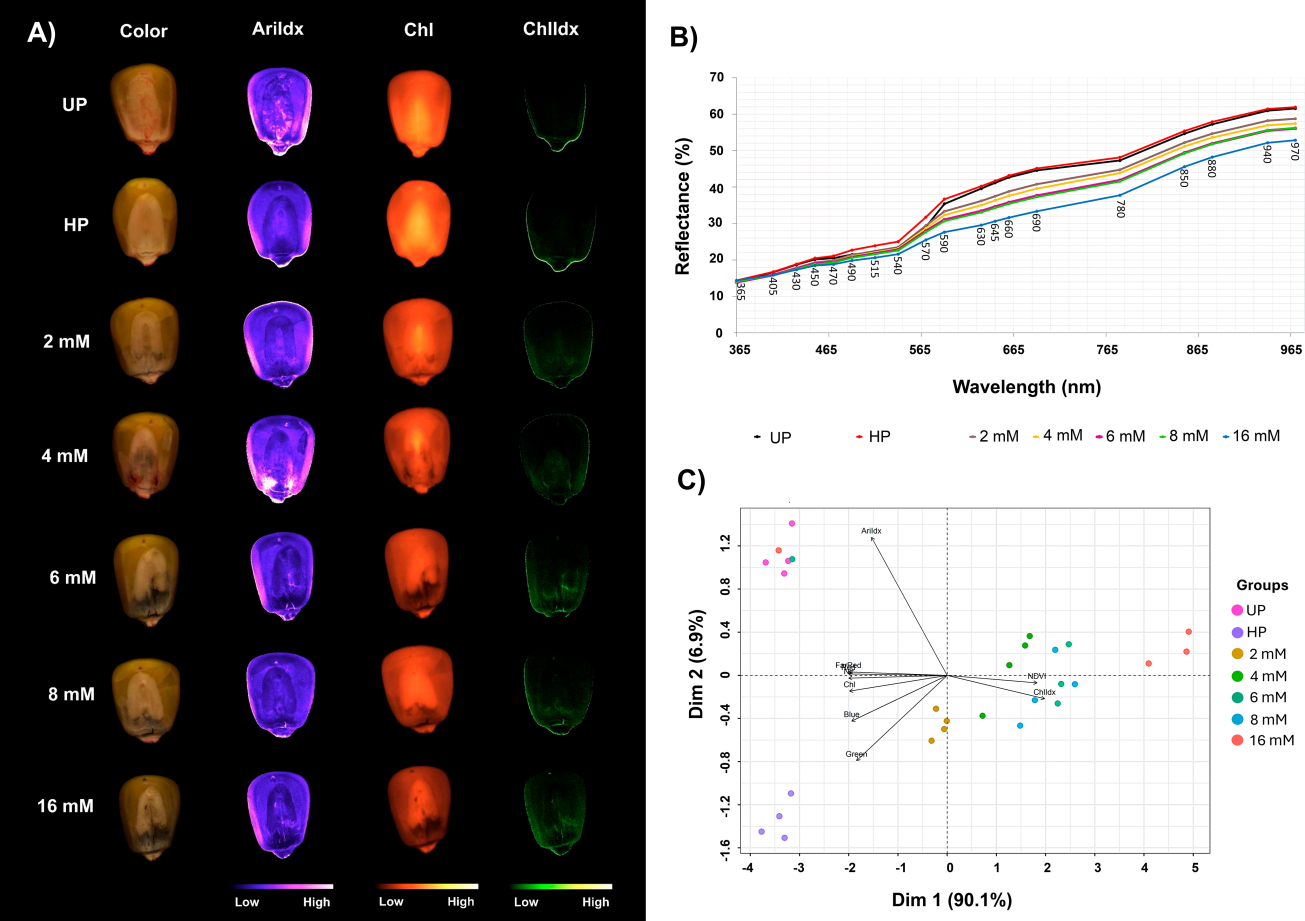
Multispectral imaging analysis of maize seeds under Bio‐NPs priming. (A) RGB (visible spectrum) images of maize seeds and corresponding images of anthocyanin index (AriIdx), chlorophyll *a* fluorescence (Chl) and chlorophyll *a* index (ChlIdx). (B) Spectral signature of primed seeds with Bio‐NPs registered at 19 different wavelengths demonstrating a reduction in the reflectance signal captured as the concentration of the Bio‐NPs increased. (C) Principal component analysis (PCA) of multispectral data obtained by SeedReporter™ from primed seeds, separating the samples into five groups, based on treatments: (i) Unprimed (UP), (ii) Hydro‐primed (HP), (iii) 2 mM, (iv) 4 mM, 6 mM, 8 mM, and (v) 16 mM Bio‐NPs priming treatments.

Our results indicated that the seed imbibition process was completed after 20 h of soaking in distilled water or nanoparticle dispersion. After conditioning, the seeds acquired a typical dark color regarding the Bio‐NPs dispersion, mainly in higher concentrations of Bio‐NPs (Figure 2A). The results of multispectral reflectance data obtained by the VideometerLab™ instrument indicated that increasing concentrations of Bio‐NPs led to a decrease in seed spectral reflectance for each emission wavelength, likely due to increased light absorption by the Bio‐NPs, confirming the enhanced seed coating by the nano‐priming treatment (Figure 2B).

The PCA method was applied to the multispectral data from seeds treated with different concentrations of Bio‐NPs dispersion. The analysis showed that Bio‐NPs seed priming could explain 97% of the total variance in reflectance (Figure 2C), with the anthocyanin Index measurement having the most impact. The grouping into three distinct classes indicates that Bio‐NPs treatments seem to be related to the number of Bio‐NPs deposited on the seed's surface. Bio‐NPs concentration of 2 mM does not impact the reflectance characteristics of the seeds, while concentrations of 4 mM, 6 mM, and 8 mM produced similar effects. The highest concentration (16 mM) resulted in the lowest reflectance among treatment conditions, likely due to the lower saturation of the seed surface. This variation in seed reflectance can be applied as a reliable indicator to monitor the progress and completeness of the priming process.

### 3.3 Physiological effects of the Bio‐NPs in maize seedlings

**FIGURE 3:**
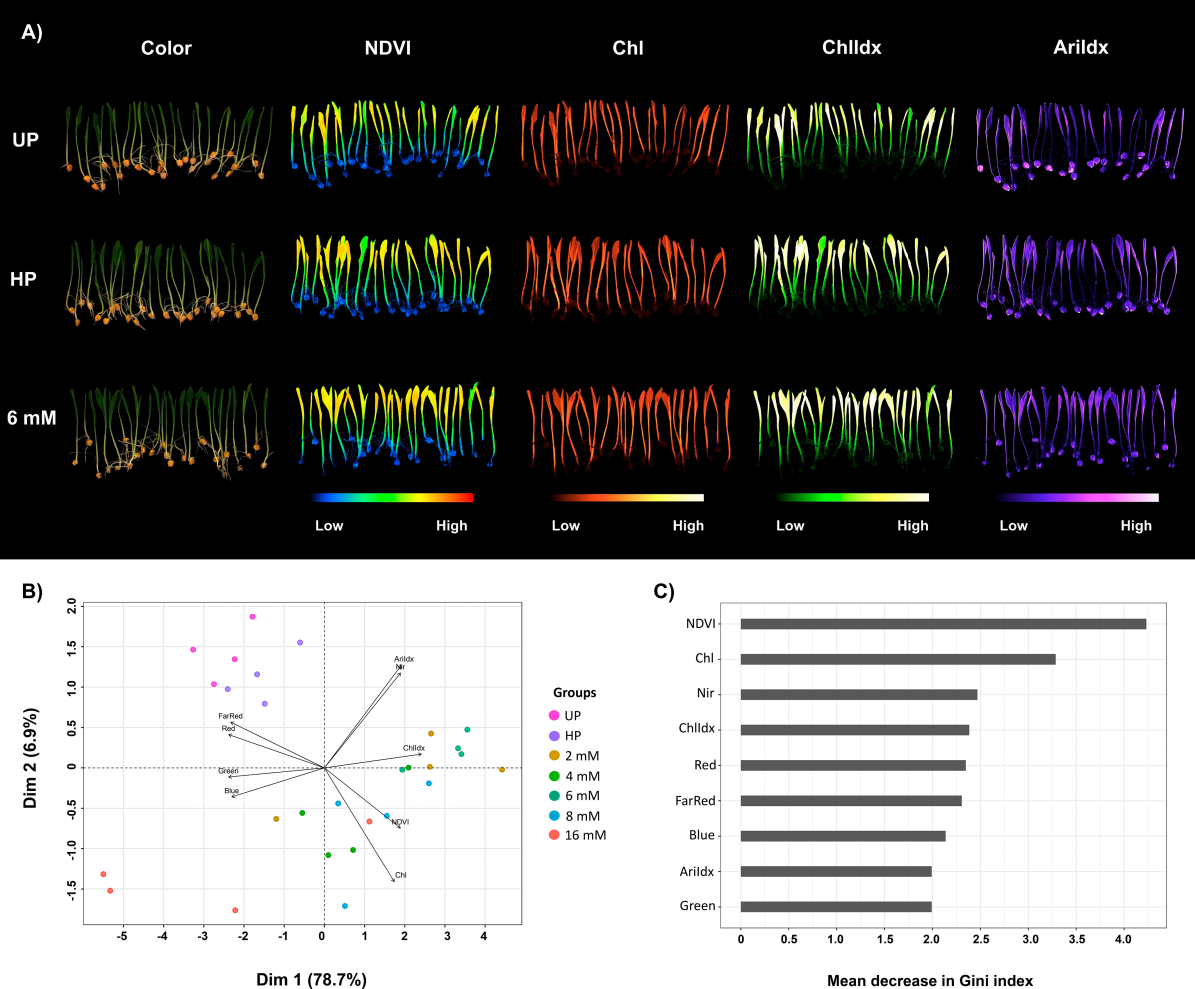
Physiological responses of maize seedlings to seed priming with Bio‐NPs. (**A**) Color images (visible spectrum) and corresponding images of normalized difference vegetation index (NDVI), chlorophyll *a* fluorescence (Chl), chlorophyll *a* index (ChlIdx) and anthocyanin index (AriIdx) captured from seedlings produced from unprimed seeds (UP), hydropriming (HP) and seeds treated with 6 mM of Bio‐NPs. (**B**) Biplot of principal component analysis (PCA) using multispectral reflectance data of seedlings resulting from UP, HP, and primed seeds with different concentrations of Bio‐NPs: 2 mM, 4 mM, 6 mM, 8 mM and 16 mM of Bio‐NPs. (**C**) Random Forest analysis to identity the parameters with the greatest contribution to the discrimination of seedlings from different seed priming treatments (*n* = 4, *p* < 0.05).

The general physiological status of the maize seedlings was investigated using multispectral imaging data, focusing on the following parameters: NDVI, chlorophyll *a* fluorescence, chlorophyll *a* Index, and anthocyanin index, which are related to physiological responses of plants (Figure 3A).

The PCA revealed that the first two principal components accounted for 85.64% of the observed variation (Figure 3B), with Bio‐NPs priming being the most impacting factor. The PCA biplot revealed three distinct clusters based on treatment dissimilarity. Most of the Bio‐NPs treatments (2 mM, 4 mM, 6 mM and 8 mM) grouped into one cluster, diverging from the 16 mM treatment, which was separated as an individual cluster with minimum principal component scores. The UP and HP treatments clustered together, separated from the Bio‐NPs treated samples (Figure 3B), suggesting that Bio‐NPs priming caused significant physiological differences in the seedlings compared to the control treatments.

A classification based on RF algorithm was applied to the multispectral data to rank the contribution of the measured features impacted by the treatments. The RF analysis indicated NDVI and chlorophyll *a* fluorescence as the most affected parameters, associated with phenotypical changes detected in the maize seedlings treated with Bio‐NPs (Figure 3C).

The multispectral image analysis revealed that Bio‐NPs priming impacted seedlings on their physiological aspects related to plant health, particularly NDVI and chlorophyll index.

**FIGURE 4:**
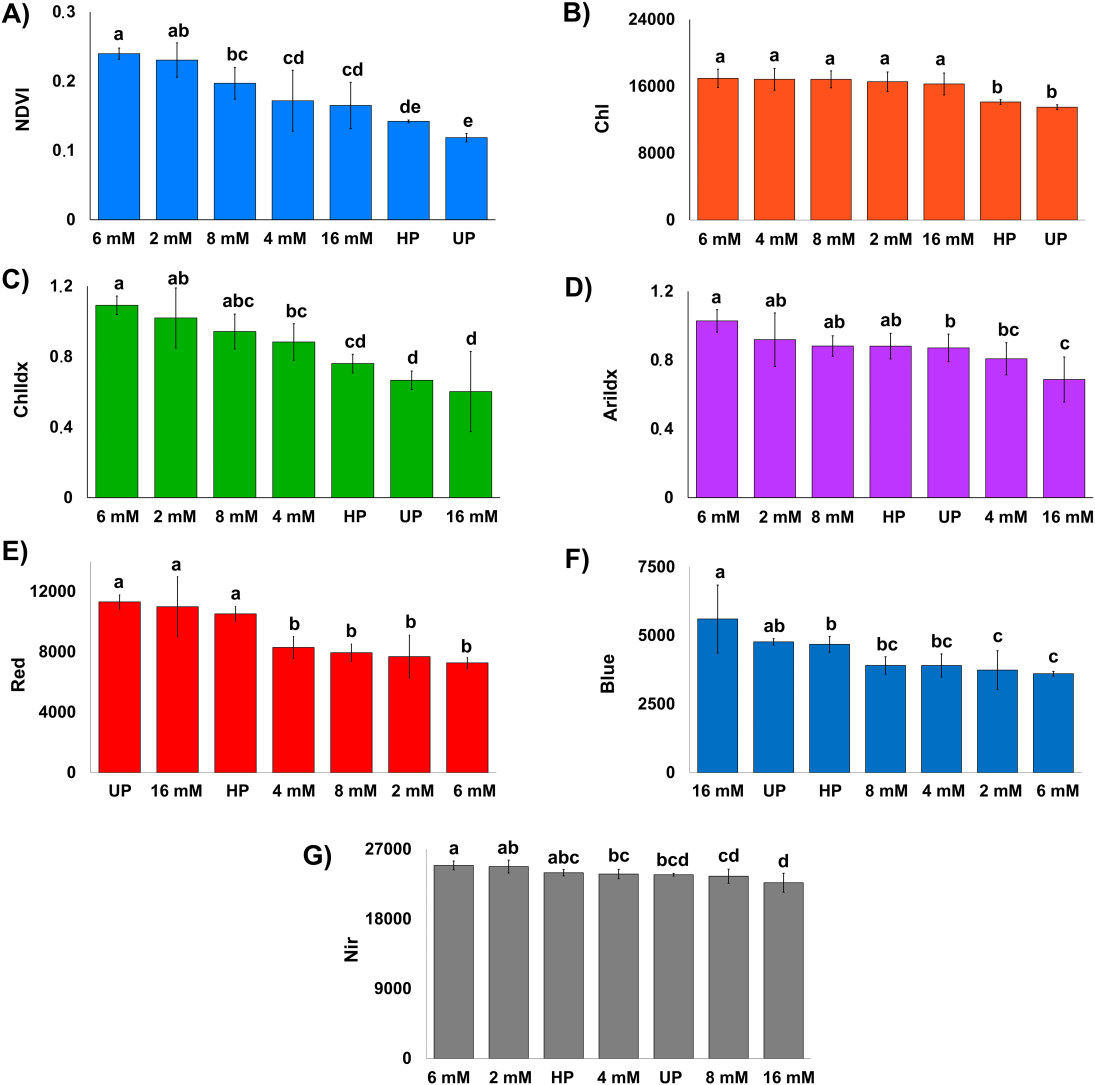
Multispectral imaging analysis of maize seedlings grown from primed seeds with different treatments with Bio‐NPs, hydropriming, and non‐priming. The bar graphics present the quantitative analysis of multispectral data for normalized difference vegetation index (NDVI) (A), chlorophyl *a* fluorescence (Chl) (B), chlorophyll *a* Index (ChlIdx) (C), anthocyanin index (Arildx) (D), and reflectance in red (E), blue (F) and near‐infrared region (G). HP and UP refer to Hydroprimed and Unprimed treatments, respectively. The indicated concentrations refer to the treatments performed with Bio‐NPs in priming treatments. The same letter indices in the bar graphics indicate mean values that are not significantly different according to the LSD test (p < 0.05). Data is expressed as mean standard deviation (SD), (n = 4).

NDVI can be implemented as an efficient tool to detect early signs of plant stress before visual symptoms manifest, functioning as a sensitive vegetation index. Developed by Rouse and Haas (1973), it measures the differential absorption of chlorophyll *a* and *b* absorbance and is an indicator of tissue structure complexity (Rouse et al., 1973). Our results revealed that seedlings from Bio‐NPs primed seeds, especially those treated with 6 mM Bio‐NPs, showed the highest NDVI values, indicating improved plant health (Figure 4A). In contrast, seedlings from UP seeds had the lowest NDVI values.

Additionally, there was a general increase in the average chlorophyll *a* fluorescence of seedlings from Bio‐NPs primed seeds compared to unprimed and hydroprimed seeds (Figure 4B). However, no differences in chlorophyll *a* fluorescence were observed between the various Bio‐NPs treatment concentrations. These increases of NDVI and chlorophyll *a* fluorescence were also verified in the images (Figure 3A).

The chlorophyll *a* index, which represents the chlorophyll content, also showed notable differences. Seedlings from Bio‐NPs primed seeds (2 mM to 8 mM) showed significantly higher chlorophyll *a* index compared to seedlings from non‐primed condition. The treatment with 16 mM Bio‐NPs resulted in the lowest chlorophyll content among the Bio‐NPs treated groups, though not statistically different from unprimed and hydroprimed conditions, indicating no toxic or detrimental effects on plant physiology. Comparing all treatments, maize seedlings from seeds primed with 6 mM Bio‐NPs showed the highest chlorophyll *a* index value.

In addition, the anthocyanin index in seedlings was assessed to understand the physiological state of the seedlings (Steele et al., 2009). There are several functions of anthocyanins in leaves, including their role in stress responses and photoprotection (Gould, 2004). The exceeding light energy received by the plants is absorbed by the anthocyanins and less light reaches the chlorophyll *b* molecules, protecting the structure of the photosynthetic apparatus under high light exposition (Gould et al., 2002).

Our findings indicated a positive correlation between increased anthocyanin content and higher chlorophyll content in seedlings treated with 2 mM to 8 mM Bio‐NPs. This suggests that Bio‐NPs priming enhances both anthocyanin and chlorophyll content, likely contributing to improved stress tolerance. The seedlings from seeds primed with 2 mM and 8 mM Bio‐NPs did not show significant differences in their anthocyanin index compared to the hydropriming and non‐priming conditions (Figure 4D). However, a significantly lower anthocyanin value was observed in seedlings from unprimed seeds and primed with 16 mM Bio‐NPs. These results demonstrated that an optimal concentration of 6 mM Bio‐NPs leads to simultaneous enhancement of both chlorophyll and anthocyanin content in the seedlings from primed seeds.

**FIGURE 5:**
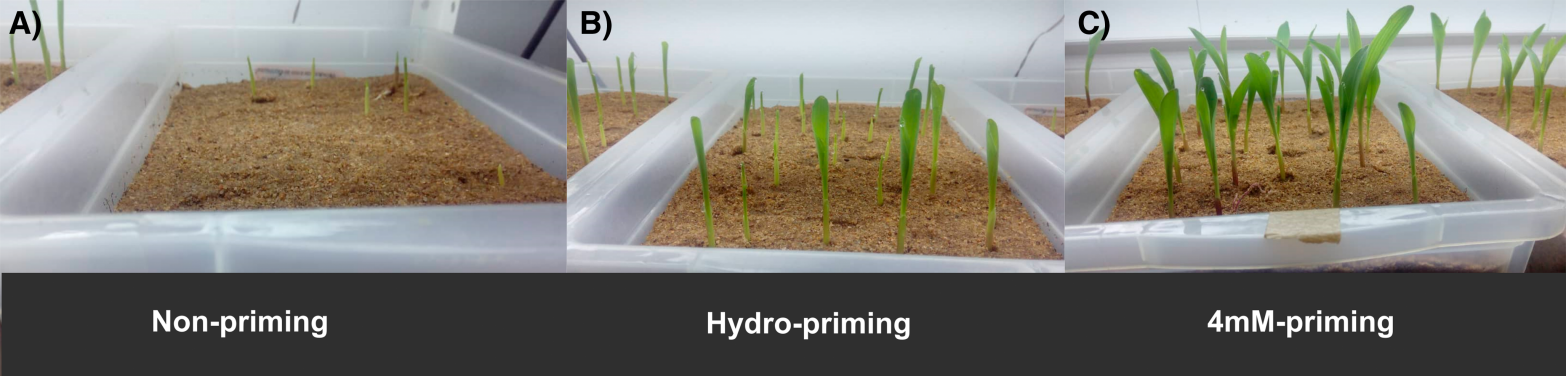
Physiological state of maize seedlings. Plant seeds that received no priming treatment (Non‐priming), hydro‐primmed (Hydro‐priming) and primed with 4 mM Bio‐NPs (4 mM‐priming) treatment were sowed in sterilized sand, and pictures were taken 7 days after sowing. Plants were cultivated in controlled 8‐h photoperiod at 25°C.

The plant seedlings treated with 4 mM Bio‐NPs showed an accelerated emergence from sand compared to HP and UP plants, indicating the potential of the Bio‐NPs as stimulants of plant emergence, which still must be confirmed in field experiments under open environmental conditions (Figure 5).

### 3.4 Effects of synthesized Bio‐NPs on microorganisms in an agricultural context

#### 3.4.1 Fungal response to synthesized Bio‐NPs exposure

**FIGURE 6:**
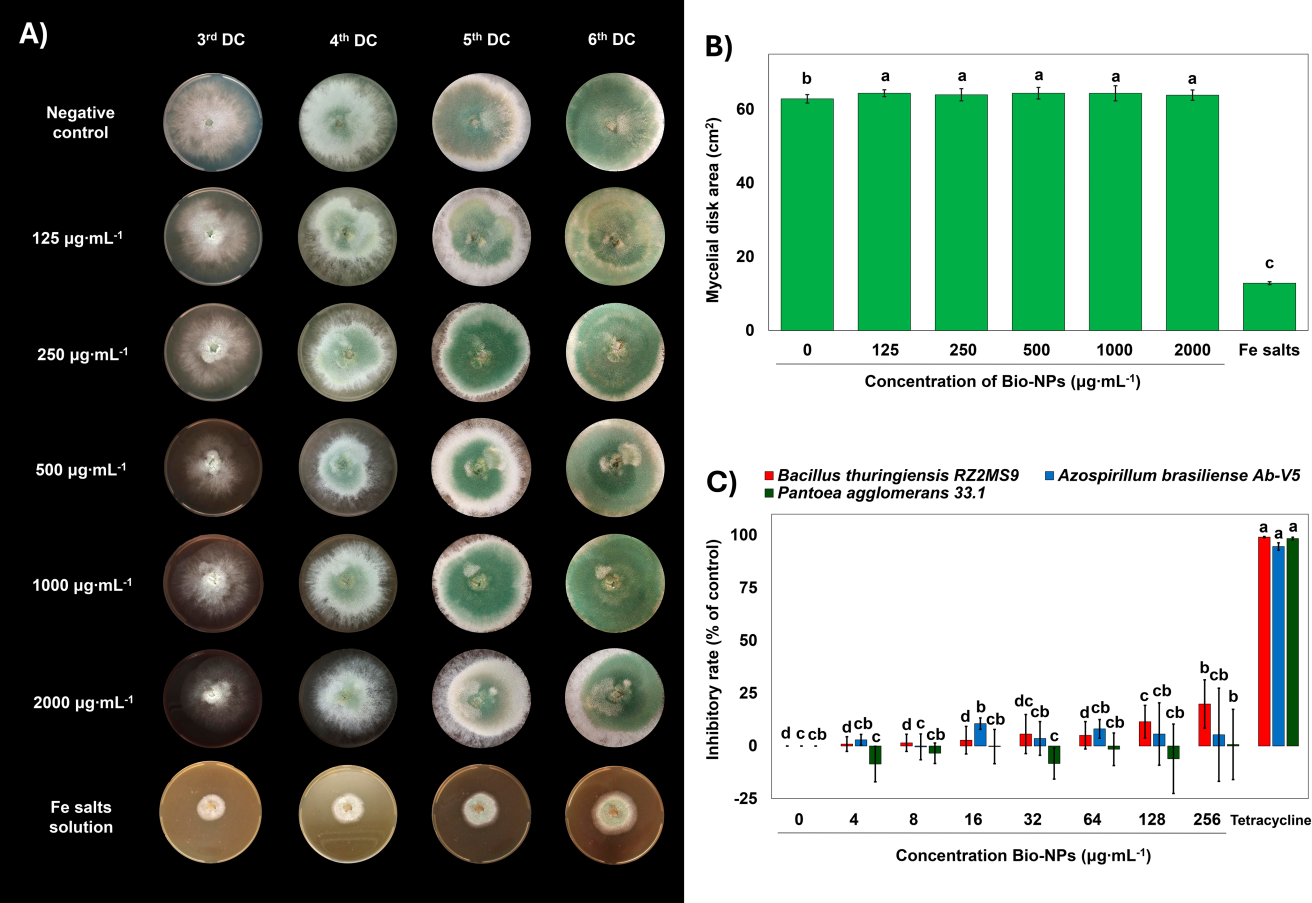
Growth inhibitory effects of Bio‐NPs on agronomically important microorganisms. (A) Fungi *Trichoderma harzianum* response to biosynthesized Bio‐NPs exposure to five different concentrations and Fe salt precursor solution of the NPs (used in Bio‐NPs synthesis), along with a negative control. Significant growth inhibition was not seen for Bio‐NPs treatments. However, Bio‐NPs slowed the mycelial growth rate at high concentrations. (B) Agar incorporation method assay. Means followed by the same letter are not significantly different according to the LSD test (p < 0.05). Data are expressed as mean and SD; *n* = 4. (C) Evaluation of different concentrations of Bio‐NPs (4, 8, 16, 32, 128, and 256 μg mL^−1^) on the growth of three bacteria species (*Bacillus thuringiensis, Azospirillum brasiliense*, and *Pantoea agglomerans*), showing low to no inhibitory effects at concentrations below 8 μg mL^−1^. Means followed by the same letter are not significantly different according to the LSD test (*p* < 0.05). Data are expressed as mean and SD; n = 4.

The impact on the growth inhibition of *Trichoderma harzianum* was evaluated at several concentrations. *Trichoderma harzianum* is a beneficial fungus used as a biocontrol agent in agriculture; thus, it is essential for developing new strategies against phytopathogens (Poveda, 2021). The agar incorporation method was used to determine the effect of different concentrations of Bio‐NPs (2000, 1000, 500, 250, and 125 μg mL^−1^) on *Trichoderma harzianum* mycelial growth. When the fungi were grown in the negative control conditions (PDA medium without Bio‐NPs), they completed their growth (covering the entire medium surface) by the sixth day of culture (Figure 6A). Interestingly, no significant difference was observed in the growth of *T. harzianum* across different Bio‐NPs concentrations compared to the control. However, when treated with a concentration of 2000 μg mL^−1^ of the original iron salt used for Bio‐NPs synthesis, fungal growth was inhibited by 52% ± 3.68%, likely due to the salt's antifungal activity potential (Figure 6B).

The results demonstrate that the molecular structure of iron affects the growth of beneficial microorganisms. While iron in the salt form exhibited antifungal properties, Bio‐NPs were highly biocompatible with *T. harzianum*, causing no phenotypical toxicity. These results are consistent with previous research demonstrating that some types of Bio‐NPs are not only non‐toxic to *T. harzianum* but can even act as a growth promoter and enhance the biological activity of *T. harzianum* (Bilesky‐José et al., 2021). Studies evaluating different concentrations of iron and copper in *Trichoderma* cultures indicate that iron plays a crucial role in electron transport, lipid oxidation homeostasis, and adenine synthesis (Tavşan and Ayar Kayali, 2013). Therefore, this report points out another interesting application of this type of Bio‐NPs as a bio‐stimulator not only for plant metabolism but also as a promising microbiome modulator, which requires further investigation.

#### 3.4.2 *In‐vitro* bacterial response under biosynthesized Bio‐NPsBio‐NPs exposure

The possible side effects of Bio‐NPs treatments on beneficial microorganisms were evaluated through their action on *Bacillus thuringiensis*, *Azospirillum brasiliense*, and *Pantoea agglomerans*. Growth inhibitory rates were calculated based on optical density relative to the control inoculum. The results indicated that increasing concentrations of Bio‐NPs had no significant effect on bacterial growth (Figure 6C). In contrast, tetracycline treatment (all concentrations) completely inhibited bacterial growth and significantly reduced cell viability, as determined by the resazurin assay. In contrast, no reduction in cell viability was observed in any microplate well treated with Bio‐NPs.

Interestingly, *B. thuringiensis* growth inhibition slightly increased with Bio‐NPs concentration above 32 μg ml^−1^, but none of the concentrations led to an inhibition rate above 25%. Therefore, the inhibitory rate observed does not indicate an inhibition phenomenon since we should have seen higher inhibition at 256 μg mL^−1^ concentration of exposure according to CLSI standards. Interestingly, for the other two bacterial strains (*A. brasiliense* and *P. agglomerans*), growth inhibition rates became negative at higher Bio‐NPs concentration (up to −21% at 256 μg mL^−1^), suggesting a potential growth‐promoting effect of these Bio‐NPs on these bacteria.

## 4 DISCUSSION

### 4.1 Effects of Bio‐NPs on maize seedlings and possible outcomes

The results revealed that the Bio‐NPs have a biostimulant effect on maize plant seedlings after seed priming, with general positive effects on plant's health and chlorophyll index and no toxic effect on the microorganisms tested, indicating their potential environmental compatibility in modern agriculture. The chlorophyll content increased in all plants treated with Bio‐NPs even though the Bio‐NPs are synthesized from iron salts that were fully incorporated into the nanoparticles. Iron (Fe) is an essential element for plant growth and cellular metabolism (Legay et al., 2012). Fe plays an important role in chlorophyll synthesis, with much of the iron in leaves found in chloroplasts (Terry and Abadía, 1986).

However, our results did not show a direct correlation between the increased Bio‐NPs concentration and higher chlorophyll content, which would be expected if the biological effect observed is related only to the iron present in the composition of the Bio‐NPs. This indicates that the modulation of the physiological and multispectral characteristics of maize seedlings is likely related to the presence of microalgae metabolic compounds constituting the Bio‐NPs, which may be influenced by the nanostructure itself.

The fact that the total chlorophyll fluorescence was only slightly lower in HP and UP plants indicates that, in general, Bio‐NPs had no detrimental effect on plant health. No evidence of stress was observed in the plants from seeds treated with Bio‐NPs, suggesting that even under high concentrations of Bio‐NPs, they caused no damage to the plants. The even distribution of chlorophyll may suggest that all plants are nutritionally healthy since leaf nitrogen is linked to chlorophyll content (Gitelson et al., 2003). The increased levels of chlorophyll *a* and chlorophyll *b* in Bio‐NPs‐treated plants have already been reported in seed priming of *Citrullus lanatus* var. Colocynthoides by macroalgae extract from *Ulva lactuca*. These effects may be associated with the presence of mild oxidative stress in Bio‐NP‐treated plants, which elicit adjustments on the central metabolic fluxes, which contribute to preventing the accumulation of reactive oxygen species (ROS) by producing protective compounds. Nevertheless, the stress‐induced responses in plants are challenging to elucidate due to their dynamic characteristics, which are subjected to variations depending on their intensity, duration, and genetic factors (Savchenko and Tikhonov, 2021).

The microalgae nanoparticles could contain basically any cellular metabolite, including phenolics, amino acids, small peptides, polysaccharides and several secondary metabolites. Considering the physico‐chemical characteristics of the synthesized nanoparticles we characterized by spectroscopic methods, the “corona” or the surface of these nanoparticles is constituted basically with organic compounds. Furthermore, considering that the nanoparticles are negatively charged, it is expected that molecules with a positive charge, such as cationic aminoacids and cationic phenolics and flavonoids, are present (Del Mondo et al., 2022). A more specific metabolomic analysis of the compounds extracted from the nanoparticles's corona would certainly help to elucidate that.

The Bio‐NPs are likely modulating the initiation of a systemic ROS signaling without being associated to the cellular damaging stages of a complete ROS stress response. From another perspective, the physical–chemical characteristics of the nanoparticles themselves (shape, charge, ultrastructure) and the metabolites in their corona may together induce an effect on the stimulation of the anthocyanin synthesis in plants by providing precursors or inducers of the anthocyanin pathway (even though microalgae has not revealed to have a cyanidin pathway per se) (Del Mondo et al., 2022).

A higher anthocyanin level could enhance light harvesting in treated plants, leading to exceeding transient ROS production, which could finally transiently activate ROS signaling pathways without the activation of photoinhibition. After the metabolization of the inducer molecules, the plants can develop to normal conditions after the transient effect induced by the Bio‐NPs. That said, the effect would be transient and effective in a dose‐dependent manner.

Moreover, the near‐infrared (Nir) reflectance measurements also indicate that Bio‐NP treatments did not negatively affect plant physiological parameters related to oxidative‐stress responses, such as chlorophyll bleaching, growth inhibition, among others (Savchenko and Tikhonov, 2021). HP and UP plants showed intermediate spectral characteristics among the Bio‐NPs treated, reinforcing that all plants were generally healthy. Seedlings from seeds treated with 16 mM Bio‐NPs had the lowest NIR values, indicating a possible reduction in the bio‐stimulating effects of Bio‐NPs at this concentration, but without signs of toxicity or damage to the physiological parameters of the plants.

The red and blue reflectance results showed that plants from seeds primed with 16 mM Bio‐NPs, as well as UP and HP plants, had higher red and blue reflectance values, while plants from 2 mM, 4 mM, 6 mM and 8 mM treatments showed lower values. Therefore, the results suggest that plants treated with intermediate concentrations of Bio‐NPs are likely to maintain leaf mesophyll structure with better osmotic responses. High blue reflectance has been previously associated with increased phenolic substances in the cell wall and/or vacuoles of plant leaves (Stober et al., 1994), while red and NIR reflectance are directly related to chlorophyll content in plant leaves (Figure 4).

**FIGURE 7:**
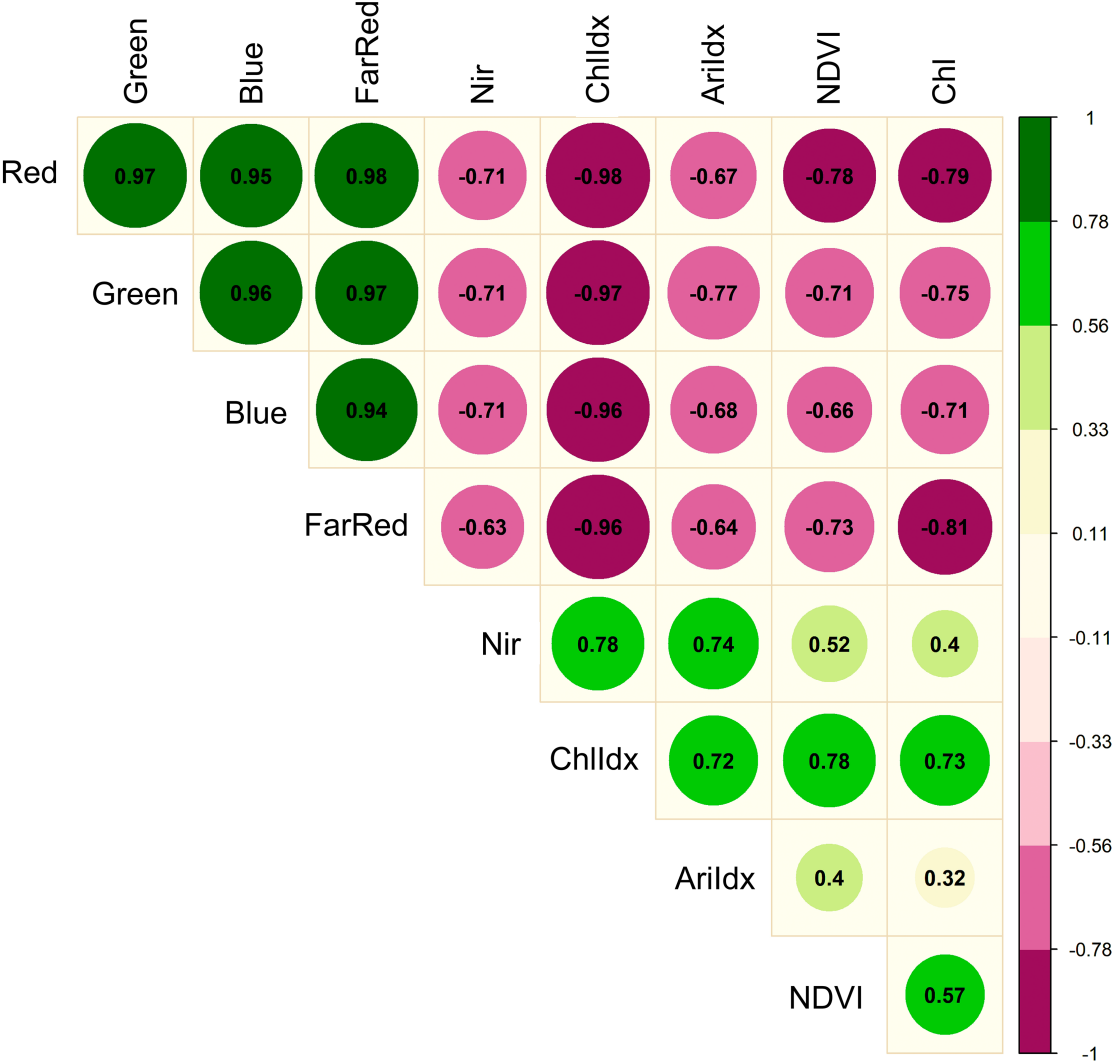
Correlation analysis between parameters obtained in multispectral imaging of maize seedlings produced from seeds treated with different concentrations of Bio‐NPs, hydropriming, or unprimed. Pearson's correlation coefficient between spectral parameters indicates that some groups present positive and negative correlations between them, systematically analyzing the correlation between chlorophyll *a* index (ChlIdx), chlorophyll a fluorescence (Chl), anthocyanin index (Arildx), normalized difference vegetation index (NDVI), as well as reflectance signals in the red, green, blue, far red, and near‐infrared (NIR) regions. The Pearson correlation coefficients between the analyzed features are provided inside the corresponding correlation circles of the image.

Healthy plants reflect from their leaves a large amount of NIR light in the 700 to 1,100 nm wavelength range. Unhealthy plants are poor in intercellular architecture and chlorophyll content, which leads to lower reflectance in NIR light (Rizk and Habib, 2018). Thus, the positive correlation coefficient between NIR and chlorophyll *a* index (r = 0.78) confirms that treated plants were not under significant stress conditions (Figure 7). Hence, an effective way to identify healthy vegetation is to measure its NIR reflectance. Regarding the NIR parameter, seed priming with Bio‐NPs at 16 mM, compared to the rest of the concentrations, reflected this energy wavelength in a similar way to non‐priming and 8 mM priming conditions, indicating no cell damage even from the higher nanoparticle concentration since there was no observed difference when compared to untreated plants. In contrast, 6 mM Bio‐NPs significantly increased NIR reflectance of seedlings compared to non‐primed treatment (Figure 4G).

The increment in anthocyanin levels in seedlings treated with 6 mM Bio‐NPs may indicate the activation of a photoprotective/photo‐abatement mechanism in the plants induced by the microalgae extract compounds present in the Bio‐NPs (Zhao et al., 2022). Leaves with higher anthocyanin content frequently exhibit traits typical of those growing in shaded environments, such as lower chlorophyll a/b ratios and higher total chlorophyll content than the green leaves of the same species (Landi et al., 2015). However, light‐independent factors can also influence anthocyanin production in plants. A previous meta‐analysis revealed that heavy metals, nutrient deficiencies, low temperatures, and UV radiation increased anthocyanin levels in plants by over 50% compared to those under normal growth conditions. In contrast, drought, high light, and salt stress raised anthocyanin levels by 41.9%, 20.8%, and 9.4%, respectively, relative to plants in standard growth environments (Yan et al., 2022).

It is, therefore, more likely that the enhanced anthocyanin content observed in our results is related to an induced plant abiotic stress response triggered by Bio‐NPs, which could enhance plant's ability to deal with the natural stress responses taking place during seed germination while accelerating the germination process by activating ROS signaling events. This would reduce the transient exceeding content of ROS and contribute to plants performing better during seedlings' emergence from soil. That transient phenotype could give plants an additional resilience towards extreme environmental conditions, providing a higher chance to the plants to quickly emerge from soil and maybe resist the climate extremes (Ali et al., 2023).

Although multispectral imaging analysis offers a non‐invasive, remote sensing technology for detecting plant biochemical and physiological parameters, it is relevant to acknowledge that plant reflectance spectra are the result of a unique combination of dynamic factors that will affect pigment absorption. These include gene expression changes, developmental stage, the presence of stressors, and environmental conditions among other factors, which may cause great variability in the plant's reflectance profile. Therefore, further molecular analysis is necessary to elucidate the specific biological processes modulated by the Bio‐NPs.

### 4.2 Implications for agricultural practices and outlook

The findings from this study demonstrate the potential of using microalgae‐derived Bio‐NPs as a biostimulant for enhancing seedling development. By leveraging eco‐friendly methods for nanoparticle synthesis, this research paves new pathways for sustainable agricultural practices that minimize reliance on synthetic fertilizers and biostimulate plants towards enhanced climate resilience. The improved physiological responses observed in primed seeds indicate that Bio‐NPs can enhance crop resilience to abiotic stresses, which is crucial in the face of climate change and environmental extremes. In addition, this research contributes to the growing body of knowledge on the application of nanotechnology in agriculture.

The Bio‐NPs could also induce a posterior benefit to maize growth and production due to the likely induction of signaling events related to the alleviation of abiotic and biotic stress by the increased stress tolerance, as has been observed in the application of microalgae biomass in Rice and Wheat. As the Bio‐NPs carry microalgae molecules, it is expected that they also function in plant growth promotion, as has already been observed in Wheat, Maize, Tomato, Spinach, and Sugar beet, among other plants (Lee and Ryu, 2021).

Even though the analysis of the Bio‐NPs in field applications and their degradation is essential for understanding the environmental impact of this material, previous analysis of the degradation of Iron nanoparticles in the soil indicated that these nanoparticles can interact with contaminant pollutants and inorganic heavy metal ions that could modify their surface, leading to aggregation and alterations in their stability, bioavailability and toxicity.

However, further studies demonstrated that such nanoparticles, while absorbing pollutants, may function as a soil remediation material. Iron nanoparticles have also been shown to bind to dissolved organic matter, which reduces their absorption of soil contaminants. Therefore, alterations of the soil humidity, pH and chemical composition can modify the oxidation state of iron nanoparticles, impacting their chemical and biological transformation in the soil. Iron nanoparticles can suffer chemical or biological oxidation in the soil, interacting with other molecules, precipitating as a metal‐nutrient‐complex or being uptake by microorganisms that need iron as an enzyme cofactor. Thus, iron nanoparticles have already been described to function in positive and negative ways in the soil environment (Tao et al., 2023).

However, of note, previous studies on the in vivo toxicity of iron nanoparticles have contributed to elucidate that these materials do not pose systemic toxicity to rats fed with these compounds, which also did not show a systemic distribution in several tissues tested, indicating potential safety to animals (Yun 2015).

Therefore, the use of biologically synthesized nanoparticles not only reduces environmental toxicity but also highlights the potential of integrating natural compounds into agricultural innovations.

The application of such Bio‐NPs in large agronomic scenario could be a future benefit to several technological sectors, not only agriculture. The *H. pluvialis* biomass has many possible applications and a high commercial market demands due to its composition rich in biocompounds of industrial interest, such as Astaxanthin. The biomass productivity of these cells ranges from 0.01 to 1.9 g L^−1^ d^−1^, depending on their metabolic state and type of cultivation method, with a production cost ranging from US$6.20 to US$40.36 depending on the cultivation medium (Shah et al., 2016; Colusse et al., 2019). The application of microalgae biomass into nanoparticles green synthesis may bring additional interest to the promising industry of nanomaterials. However, the scalability of nanoparticles production and functionalization still faces many challenges to warranty reproducibility and economic feasibility, even applying emergent technologies (Paliwal et al., 2014).

The positive effects on biological soil fertility suggest that Bio‐NPs may foster a healthier soil microbiome, further promoting plant growth and soil quality. Finally, future studies should explore the following avenues: (I) long‐term effects by investigating the long‐term impacts of Bio‐NPs on plant growth, yield, and soil health, particularly under varying environmental conditions and stressors; (II) mechanistic studies conducting detailed analysis to elucidate how Bio‐NPs interact at the molecular level with plant cells and soil microorganisms, even in long‐term microbial community dynamics, since understanding these interactions can inform the design of more effective and environmentally safer biostimulants; (III) expand the research to include other crop species and different types of nanomaterials. This will help determine the universality of the benefits observed in maize and assess the applicability of these findings across various agricultural contexts; and finally (IV) field trials: transition from controlled experiments to field‐grown crops to evaluate the practical effectiveness of Bio‐NPs in real‐world agricultural settings. This will help validate laboratory findings and assess economic viability.

## 5 CONCLUSION

In summary, our results demonstrate that the Bio‐NPs can be synthesized using an environmentally friendly method using the *Haematococcus pluvialis* extract. The results of this study provide valuable insights into the effects of Bio‐NPs as a seed priming agent on maize seedlings, as well as their non‐toxic influence on agriculturally important microorganisms.

Bio‐NPs had no significant inhibitory effect on bacterial growth and even promoted growth in some cases. These findings contribute to potential applications of Bio‐NPs in agriculture, specifically in seed priming and plant biostimulation. However, the molecular effects of these Bio‐NPs must be further investigated in plant seedlings to explore the molecular effects of Bio‐NPs on seedlings to validate these findings in field conditions towards improving sustainable agriculture practices. In conclusion, as the use of nanomaterials in agriculture increases, it is paramount to understand their role in plants and in the environment. In addition, it is essential to establish clear regulatory frameworks for nanotechnology applications to ensure safety, protecting human health and the environment. Collaborative efforts among researchers, policymakers, and industry stakeholders may facilitate the development of guidelines for the application of nanotechnology in agriculture.

## AUTHOR CONTRIBUTIONS

FVW, NR, HAA, SAK and LLL: conceptualization, writing ‐ original draft, writing ‐ reviewing & editing. NR, HAA, LL: investigation, methodology of bionanoparticle synthesis and microalgae cultivation. NR, HAA, LLL, CBM, FVW: methodology of plant multispectral imaging analysis, validation, formal analysis, investigation, data curation. NR, HAA, WYH, FVW and LLL: data visualization, statistics, writing ‐ original draft, writing ‐ review & editing. LLL, LFPO, GLPG, SPL: methodology with microorganisms. MLR, APM: methodology of TEM of bionanoparticles. APM, CBM, SPL, JL: supervision, funding acquisition, reviewing & editing. FVW: supervision, funding acquisition, project administration.

## FUNDING INFORMATION

This work was financially supported by the University of São Paulo (PIPAE Grant Proc. 2021.1.10424.1.9), The São Paulo Research Foundation ‐ FAPESP (Grants 2016/06601–4, 2018/03793–5, 2018/21489–1, 2019/17721–9, 2022/15431–6 São Paulo Research Foundation ‐ FAPESP), National Council for Scientific and Technological Development ‐ CNPq (Grant number 421447/2023–0), and Coordenação de Aperfeiçoamento de Pessoal de Nível Superior ‐ Brasil ‐ CAPES (Grant number 001).

## SUPPORTING INFORMATION

Not applicable.

## Data Availability

Data sharing is not applicable to this article as all newly created data is already contained within this article.
